# The Long Fox Lecture: The Relation between Chemistry and Medicine

**Published:** 1923-07

**Authors:** F. Francis

**Affiliations:** Pro-Vice-Chancellor and Professor of Chemistry in the University of Bristol


"Scire est nescire, nisi id me
Scire alius sciret.

JULY, 1923.

### THE LONG FOX LECTURE:

DELIVERED IN THE UNIVERSITY OF BRISTOL ON MARCH 27TH, 1923.

T. LOVEDAY, M.A., *Vice-Chancellor, in the Chair.*

BY

PROFESSOR F. FRANCIS, D.Sc.,

*Pro-Vice-Chancellor and Professor of Chemistry in the University of Bristol.*

ON

### THE RELATION BETWEEN CHEMISTRY AND MEDICINE.

THE practice of Medicine—in so far as it is strictly scientific—is based on the principles established by Physiology, Pathology and Pharmacology, and the history of these shows that they have progressed as their generalisations came to be based upon the experimental results of Biology, Chemistry and Physics.

The great and complex problems of Biology which lie at the foundations of Physiology and Pathology are being resolved slowly into factors studied with the aid of the Atomic Theory and the Laws of Energy.

It seems to me, indeed, as though all other lines of research and avenues of progress in Medicine during the past fifty years have become comparatively deserted for those where advances are made through the technique and theories of Chemistry.

This period has seen a forbidding and difficult field of chemical research explored by the great chemists and physicists of the times, and I propose to place before you the main lines of thought which have brought about a profound change in the scientific aspect of Medicine.

The beginning of the nineteenth century saw the establishment of the conception of the Indestructibility of Matter, experimentally tested by Landolt in 1893, and shown to be a statement of fact to a degree of accuracy of one in ten millions. The study of that part of matter called Chemical Compounds, characterised by the constancy of their composition, led Dalton in 1808 to resuscitate the old Greek doctrine of Atoms advocated by the school of Leucippus.

Gay-Lussac and Humboldt in 1805 had investigated the composition of Steam, and discovered the extremely simple laws of gaseous combination. Six years later Avogadro, and eight years later Ampère, had interpreted these laws by a modification of the Atomic Theory, the assumption of Atomic complexes—called Molecules—and the resulting confusion of thought was only cleared up by Cannizaro in 1858.

At a date when Dr. Long Fox must have been losing interest in Chemistry, owing to the pressure of purely medical work, the broad basis of the science as it is known to-day was established.

The study of Chemical Compounds, interpreted by means of this wonderful conception of Atoms, dominated Chemistry to the exclusion of everything else for the remaining part of the century.

Another great landmark in Science, corresponding to the discovery of the conservation of Mass at the beginning of the century, was the realisation by Meyer in 1842 that energy, like matter, was indestructible. Known as the First Law of Thermodynamics, it was placed on a firm experimental basis by Joule in 1843.

This principle, however, gives no information as to the direction in which a given process takes place, and the solution of this aspect of the problem was made by Clausius and Lord Kelvin in 1850, who framed the Second Law of Thermodynamics. These laws, developed mathematically, formed a non-hypothetical system of reasoning called Thermodynamics, which dominates Physical and an ever-increasing part of Chemical and even Biological science.

Chemical and Physical phenomena can be interpreted by means of the Atomic and Molecular hypothesis, extended by the Kinetic Theory of Matter, but the results arrived at may be open to question, because the assumptions used in these theories may not be justified. But, on the other hand, there is this alternative method of Thermodynamics, against which no such criticism can be made. This method is independent of any conception of the constitution of matter, or of any physical picture one may like to adopt of the processes at work.

Down to the period—and to fix a date let me say the early sixties—Chemistry had the marvellous weapon of the Atomic Theory ready for the wonderful victories made during the remainder of the century, and a potential weapon, Thermodynamics, the use of which towards the end of the century introduced the conception of continuity and revolutionised the science.

I will trace, to begin with, the Atomic-Molecular Theory of Matter; and there is not sufficient time at my disposal to discuss those brilliant and fascinating discoveries of the last twenty years, which have completely altered our conception of the nature of the Atom.

For a century that word had meant to chemists the limit of matter. By a variety of completely independent methods physicists had counted the number of these Atoms in a given space; the agreement between the results was so striking—numerically varying but little from the errors involved in taking the census of any large city—that the belief arose that matter was really composed of Atoms, and that it was no longer necessary to look upon the conception as merely a useful working hypothesis.

Then came the discovery of Radiation, the realisation of the existence of an intra-atomic world; the Atom was no longer the limit of matter, and we can now state that "Nature uses the same standard bricks in the construction of the Atoms of all elements, and that these standard bricks are the primordial atoms of positive and negative electricity, Protons and Electrons."

The wonder, beauty and magic of these discoveries are so great that I hope a scientific Milton of the future will arise to describe them for the benefit of that great part of intellectual mankind which is ignorant of Science. This work forms the most majestic monument to the human intelligence that the world has yet seen.

The outcome of the application of the Molecular Theory to Chemical compounds was the very simple conception of Valency, that the atoms were capable of holding together a definite number of other atoms. Hydrogen being taken as unit, Oxygen could combine with two of these atoms, Nitrogen with three, Carbon with four; this enabled pictures to be drawn of the architecture of the Molecule, and upon this conception rose the edifice of Organic Chemistry.

This branch of the science commenced with the study of the products of Animal and Vegetable Metabolism. It had been freed from the incubus of Vital Force, supposed to be essential for their preparation, by the artificial production of Urea by Wöhler in 1828; but it was soon realised that the known methods were insufficient to cope with these highly complex substances, and their study was dropped for fifty years.

In the meanwhile Synthetic Organic Chemistry advanced, with the result that at present something like 100,000 new substances are known, their physical and chemical properties have been correlated with molecular architecture; a completely new technique has been invented, and a battery of weapons elaborated, which have enabled chemists during the past thirty years to take up again the study of those highly complex compounds, which play an all-important part in the activity of the living cell.

Throughout this period of some sixty to seventy years the conception of Valency has been the guiding star, offering an explanation—often uncanny—for all the curious phenomena met with. Its extension in 1874, by Le Bel and Van't Hoff, from two to three dimensions opened up new fields of thought, and a new chapter of Science was commenced, fraught with great importance to Medicine in the future.

Before I pass to those recent achievements so intimately connected with Biology let me remind you of some of the old victories. The molecular architecture of Alizarin was determined by Grebe and Leebermann in 1867; as a consequence it was synthesised in the following year, and before 1873—manufactured on a commercial scale—it had ousted the natural product from the markets of the world.

Between 1865 and 1882 Von Beyer, in Munich, had been engaged in investigating some of the most subtle problems in Organic Chemistry, and in the course of his work synthesised Indigo Blue and determined the arrangements of the atoms in the very complex molecule of this substance. Before this material could become an article of commerce yields had to be increased, and new methods of preparing intermediate products had to be devised. Time, skill, and an appreciation of the value of Science resulted in Synthetic Indigo from the Rhine factories replacing the natural product from our possessions in the East.

Ladenburg in 1886 effected the complete synthesis of Conine, one of the alkaloids of Hemlock, identical in all respects with the natural product; it was the first of the many striking feats of the extension of the conception of Valency to three dimensions.

From the study of Synthetic Organic Chemistry arose the industries of the dyes, perfumes, drugs and high explosives.

I have now come to that period—let us say the late eighties—when the accumulated experience of Synthetic Organic Chemistry had enabled chemists again to take up the study of those complex compounds so intimately connected with all life processes.

It was at this period, however, that a group of chemists with conceptions borrowed from Physics, were throwing a completely new light on chemical processes; but it will, I think, be more convenient if I follow the work of those distinguished organic chemists who achieved so much by the application of the old conceptions of molecular structure to the substances around which the phenomena of life are manifested.

I can continue this sketch because fortunately the discoveries of the current century have not yet seriously disturbed the principles underlying systematic Organic Chemistry.

The problems facing the chemist who studies the products of animal and plant life are so extremely difficult and complex that it is only the master of his craft who can isolate here and there a specific problem among the multitude, and to that problem apply the knowledge of to-day.

The starches, fats and proteins of our food, all insoluble in water, are smoothly and rapidly rendered transmissible to the blood, and it is only up to a point that we can follow this progress by comparison with similar reactions, which can be carried out in the laboratory. But following such analyses, synthetic processes are at work, the mechanism of which we are in total ignorance. A true knowledge of metabolic processes can only be obtained by the tedious unravelling of the complex system of chemical changes into individual chemical reactions. We only know a very few of these simple reactions, but there is every hope in the future that we may be able to construct an accurately itemised account of the chemical transactions of the animal body—anabolic and katabolic. The value of such knowledge for the advancement of Medicine is obvious.

The day has long since passed when the statement that—fats and sugars are oxidised in the body to carbon dioxide and water and proteins give urea in addition—is considered to be an all-sufficient explanation of the chemical role of the substances in the animal economy.

The Enzymes produced in the cells have not been synthesised by the chemist, in fact the great majority have not yet been isolated in purified form. Willstätter, however, is publishing a series of papers on Enzymes from vegetable sources and most valuable results have been obtained.

**The Proteins.**—The proteins are among the most complex substances produced in the animal and plant world. They occupy a unique position in the activities of all living matter, and a complete comprehension of their nature must obviously precede the full development of Bio-chemistry. Even their isolation in a state of purity is difficult, and their present classification, certainly of physiological importance, is little more than a monument to our scientific ignorance of this extraordinarily important group of substances.

On hydrolysis they break down to relatively simple substances, the so-called Amino Acids, and as a result of the investigations of a century some twenty of these acids have been isolated, their molecular architecture determined, and their actual synthesis accomplished in the laboratory.

It is believed that all the various proteins are built up of some or many of these twenty acids, and it was to the genius of Emil Fischer that we owed the wonderful attempt to use these substances, and from them reconstitute the original protein material; it met with but partial success, and up to the present no synthesis of a protein has been effected. Not only has it been found impossible to effect a synthesis, but the actual analysis of the great majority of protein substances has defeated the chemist. We have been unable to devise a method of determining the total amount of the various Amino Acids, resulting from the degradation of the protein. We know that some are rich in one acid, others in another, and by feeding experiments on animals much valuable information has been obtained—enough to put the whole question of Protein assimilation in an entirely new light, and to show the incalculable advantage to Medicine of complete knowledge of this subject.

Upon a knowledge of the Proteins, constituting the major part of living protoplasm, depends in all probability a knowledge of the Ferments or Enzymes, for it seems probable that proteins are the material from which the organism prepares these wonderful agents.

There is another aspect of the chemical study of the Amino Acids, which appears likely to prove of the highest importance to Medicine. Such Amino Acids as Phenylalanine, Tyrosine, Tryptophane and Histidine are always present in the intestines ; they are the harmless breakdown products of the proteins, but when these are acted upon by certain micro-organisms, capable of breaking off carbon dioxide extremely toxic substances result. It has been suggested that this may be the right direction for the chemical investigation of alimentary toxæmia and its alleged consequences, such as arterio-sclerosis and chronic renal diseases. If such toxic bases are generated in sufficient amount and taken into the blood in quantity too large for transformation (by the liver and other defensive organs) into less harmful derivatives, they must inevitably manifest their pharmacological and toxic properties. One of these bases, probably formed from Histidine, is said to stimulate the isolated uterus to contraction in the almost unbelievable dilution of one in 250 millions.

**Nucleic Acids.**—The Nucleins and Nucleo-proteids are important constituents of cell nuclei, and some extremely interesting work has been carried out during the past forty years on Nucleic Acid, a constituent of the latter material.

May I remind you of the isolation in 1868 of Nucleic Acid from pus cells, followed in 1874, by the discovery that the spermatozoa heads of the Rhine salmon consist almost entirely of Protamine Nucleate, and that this must have arisen, not directly from food, but from muscle protein. These acids obtain the Carbohydrates and Phosphoric Acid required by their syntheses from the nourishment on which the organism thrives, but do not owe the purine factor to the same source. In other words, the tissue must have the power to synthesise the purine nucleus, but we are still ignorant of the mechanism by which this synthesis is effected.

The transformations undergone by Nucleic Acid in contact with tissue extracts—that is, in the presence of Enzymes—have been studied for over thirty years, and we now have some idea of the smoothness and ease with which the living body converts Nucleic Acid into Uric Acid, and of the localisation and distribution of the Enzymes which bring about such changes.

I have no time to outline the investigations that have been carried out with such great success on the Pigments of Blossoms and Fruits, but I cannot refrain from drawing your attention to that on Chlorophyll and Hæmoglobin, which culminated in 1914.

It is to the genius of Willstätter that we owe the discovery of the chemical relationships between the two ; for an assemblage of substituted Pyrrol nuclei constitute the basic architecture of the blood pigment and of Chlorophyll, iron playing the essential part in the former Magnesium a corresponding role in the latter.

I am going to say little on the question of Synthetic Drugs. When we consider the enormous number of substances synthesised during the past fifty years, numbering certainly something like 100,000, it is disappointing to have to record the few that have been found to possess pharmacological value, or certainly novel pharmacological reaction. A substance like Salvarsan is a brilliant exception. Innumerable changes have been rung on such groups as Local Anæsthetics, Hypnotics and on Salicylic Acid, valuable substances have certainly been found, the dependence of physiological action on molecular architecture has been explored and much valuable work accomplished ; they have not led, however, to discoveries of outstanding importance to Medicine. But in the actual synthesis of substances, such as Adrenalin (from Supra-renal gland), and Thyroxin (from the Thyroid), the organic chemist has been of great assistance to the physiologist and the physician in his examination of the endocrine apparatus. The replacement of dried glands by the pure substance itself means that the dosage is much more exact and the effects more to be depended on.

I have sketched very briefly the progress of knowledge during the past fifty years, based on the conception of Valency; the success in this domain has been so great that many are convinced that we are masters of these problems, and that in the course of time we shall be able to prepare synthetically any product of Nature. But I am inclined to think that a halt has come in this progress, and that we now await the discovery of the methods employed by Nature, so very different from those used in our laboratories. With the discovery of these methods a new Organic Chemistry will arise, the importance of which to Medicine, nay, to mankind in general, will be immeasurably greater than the old.

Up to this point I have pictured the application of Chemistry to the problems of Biology from one point of view only, namely, that of Molecular Architecture, and indicated the attempts that have been made to determine the structure of those complex substances, and the further problem of their actual synthesis, the second complementary to the first.

But there was one aspect of this general problem entirely omitted from consideration; that is, the question of Energy. Since no chemical reaction takes place without energy changes, it is clear that this factor is equally important, nay, essential for the full understanding of the reactions of the living cell.

As a theoretical weapon the Molecular Atomic conception of matter is incapable of assisting us in the interpretation of energy changes, but I have already mentioned that the system called Thermodynamics, independent of any theory of matter, and elaborated in the early fifties, was ready for use in the realms of Chemistry.

Willard Gibbs, Professor of Mathematics at Harvard, showed most clearly in 1874 the possibility of the application of Thermodynamics to chemical phenomena. His work marked an epoch as important as that of Lavoisier, and it is not going too far to state that Gibbs is the greatest scientist that America has yet produced. Gibbs's treatment of the subject was mathematical, and chemists, as a rule, were non-mathematical; and it was not until the eighties that Van't Hoff and many others used this process of reasoning as one of the ordinary weapons of the science.

And at this stage there appeared that unfortunate name of Physical Chemistry; and again in order to date episodes I shall give you the year 1890, in which Sir J. Walker published the translation of Ostwald's *Outlines of General Chemistry*, and familiarised English chemists with this new method of treating chemical phenomena.

I use the word "unfortunate" advisedly, for it led the uninitiated to think that it was a new branch of Chemistry, when it was nothing more than the application of new methods to the phenomena of the science. It looked upon these phenomena with new spectacles, and these new spectacles revealed a new world, and literally gave birth to a new Chemistry.

The study of chemical reactions, interpreted by Guldberg and Waage between 1864 and 1879, by means of the Kinetic Molecular Hypothesis, had resulted in the recognition of one of the most fundamental principles of Science—the so-called Law of Mass-action. The relative mass of substances present in unit volume entering into a chemical reaction influences the proportion of the various products of that reaction. Reactions may cease before all the substances on one side are converted into substances on the other—as we would represent the reaction in a chemical equation. The whole system comes into a state of what is called Chemical Equilibrium. A reaction, indeed, may be reversed by changes in the concentration of the reacting bodies. The law was deduced, the effect of varying the amounts of the interacting substances could be calculated, but nothing was revealed as to the effect of altering the temperature, nothing as to the energy that could be obtained from such a reacting system.

The application of Thermodynamics to this problem gave the complete picture, and showed that Guldberg's and Waage's discovery was but a special case of a general theorem.

Here, then, was a new weapon for the investigation of chemical reactions and for the study of chemical affinity, and the application of these conceptions showed that the forces which bring about Chemical reactions and those which bring about the so-called Physical reactions, such as Solution, or the change of ice into water or water into steam, are all subject to the same laws and can be measured in the same way. With the study of equilibrium the idea of continuity entered Chemistry and transformed the science. And although we have certainly no such simple and universally important law such as Newton's for all the complexities of chemical phenomena, we do possess a number of universal natural laws which make possible a formal description of the course of a reaction.

Until the introduction of these conceptions Bio-Chemistry consisted of a great number of descriptions of different products accompanying living organisms, their properties, their percentage composition, in some cases their use, in some few cases the arrangement of the atoms in their molecules. But even this quantitative element—the determination of the relative amounts of the elements present—was lacking in cases where the substance investigated occurred in such small amounts that isolation in the pure form was impossible.

Unless the newer methods of Chemistry—to which I am now alluding—be employed, the old classical science could but indicate the occurrence of such substances, their mode of preparation in the most concentrated and purest possible form, and in such condition enumerate their characteristic properties.

But the new methods made it possible to form an opinion on the manner in which they react and thereby gain a clear scientific insight into their nature.

Remember that most of those substances, such as Enzymes or Toxins, are so unstable that their solutions cannot be heated to 60°C., that in many cases they are destroyed by acids or alkalis, and that often they cannot be separated from the albuminous substances which accompany them. The new Chemistry allows us to follow quantitatively the influence of temperature, or of foreign substances upon these interesting organic products of vital importance to the physiological processes of daily life, in disease and in therapy. The value of such methods in that branch of Medicine called Immune-Chemistry must be obvious.

The influence of temperature on the velocity of different processes in which Enzymes take part, or organic products such as egg white, or living cells such as blood corpuscles, bacilli, or such higher organisms as eggs or plants, has been proved to follow the same law as is found for the influence of temperature on ordinary chemical processes.

The quantitative laws of Chemistry have been found to hold when life processes in which not simple cells, but higher organisms are involved—as, for instance, in the experiments on the digestion of food by dogs. The data regarding digestion, secretion and resorption in an animal's body show a great number of very simple regularities, the existence of which in such " vital " processes—which depend to a high degree on psychical effects—was deemed impossible.

Experiments with trypsin, diphtheria toxin and antitoxin, cobra poison and its antibody, again show that the same laws are valid for the equilibrium in which antibodies and antigens enter as for the equilibria studied in general Chemistry.

The quantitative determination of those equilibria leads to the conclusion that the antibodies are not analogous to enzymes or catalysts—as was often maintained—but really take part in the equilibrium.

The study of such actions throws much light on the progress of illness produced by micro-organisms, and it is a promising feature that the content of antibody during and after illness can be treated in a strictly quantitative manner, and that it has been found possible to subject this extremely important phenomenon to calculations which agree as well with the observed facts as with ideas conceived for the explanation of other parts of natural science.

The new methods enabled us to investigate chemical processes in which simple cells—such as yeast cells, blood corpuscles or bacteria—act upon or are treated with chemical reagents, processes such as fermentation by yeast cells, hæmolysis by means of hæmolytic poisons, the agglutination of bacilli by agglutinins, and the killing of bacilli by those poisons called disinfectants. All these are processes with which the old classical Chemistry was incapable of dealing.

**Solutions.**—Many of the phenomena resulting from the solution of substances in a pure solvent were discovered, lost and rediscovered several times during the latter part of the eighteenth and early part of the nineteenth centuries. But it was not until much later that Van't Hoff, in 1887, offered an explanation of the phenomena in terms of the Kinetic Molecular Theory and of Thermodynamics.

You will remember that according to Avogadro equal volumes of gases at the same temperature and pressure contain the same number of molecules; or, any kind of molecules enclosed in equal volumes show the same pressure at the same temperature.

Van't Hoff extended this conception to solutions; all dissolved substances produce upon a membrane which prevents their diffusion, but a membrane through which water can pass, an Osmotic Pressure equal to that which would be produced by gaseous matter containing the same number of molecules in the same volume. Or, in solution the same number of molecules of any kind of matter produce at the same temperature and volume the same pressure on the walls which prevent their diffusion.

Living cells are surrounded by such walls—semi-permeable diaphragms—and Osmotic Pressure regulates the exchange of water between cells and tissues and the liquids of the animal body.

This means that the exchange of water between the tissues and liquids of the body can be expressed by Van't Hoff's theory of Solution, that a typical life phenomena is reduced to a fundamental chemical law.

There arises the extremely interesting question of dead and living cells considered as semi-permeable membranes. The living cells of the kidney can concentrate urea from 0.04 per cent. in the circulating blood to 2 per cent. in the urine. Such cells, in fact, appear to act in the opposite manner to dead semi-permeable membranes, and there are those who draw the inference that here there is a form of energy and energy transformer at work which has not been observed elsewhere than in living cells.

Closely connected with Osmotic Pressure of Solutions, and capable of being calculated from it, but much more easily determined experimentally, are (1) the lowering of the freezing point, (2) the rise in boiling point, and (3) lowering of vapour pressures.

Our conception of the nature of Solutions was extended by Arrhenius in 1888, as a result of the investigations made on the passage of electricity through solutions, and of a study of Osmotic Pressure of such solutions, together with these related phenomena.

These substances, which on solution enable that solution to conduct electricity, consist not of molecules, but of molecules broken down into highly-charged atoms, or groups of atoms, termed Ions ; the extent of this breakdown can be calculated from the conductivity of such solutions, by the determination of their Osmotic Pressure, or by those other related phenomena I have mentioned, or indeed by purely chemical considerations.

All acids showed one set of properties in common, because they all give rise to the same thing—hydrogen ions—when they are dissolved in water. All bases turn red litmus blue, because when dissolved they all give rise to hydroxyl ions. The old methods of the science were incapable of determining the amount of these present, that is, the real acidity or basicity of a solution. The amount of an acid or base present in a solution as determined by titration has nothing to do with the acidity or basicity of that solution, that is, its " PH."

Water does not conduct electricity ; dissolve salt in it, and it does ; the Osmotic Pressure of the solution, the rise in its boiling point, the lowering of the freezing point, all show that there are two things present ; the molecule of

Salt has broken down to give ions of Sodium and Chlorine, and according to this conception these, not the molecules, are the carriers of the current. All chlorides give the same test for Chlorine, because they all give rise to the same Chlorine ion. And note, the extent of the breakdown of the salt molecules can be calculated by the conductivity of the solution. Here was an entirely new point of view; chemical activity could be estimated by electrical methods.

The importance of this completely new outlook with regard to the nature of solutions was clearly of profound importance to physiologists. The body consists of over 63 per cent. of water and the cell protoplasm of 80 per cent. The materials for the nutrition of the cells of the body are conveyed to them in aqueous solution; the chemical reactions which proceed in the cells take place in the same medium. The worn-out material, the result of cell action, is carried away in solution; but there is no need to emphasise the importance of this subject. I believe I am right in stating that the phenomenon of life is unknown apart from solutions.

**Colloids.**—The further study of solutions revealed a new type, a new subdivision of matter termed Colloidal.

Albumen, for instance, is soluble in water, but the solution shows such an extremely small osmotic pressure, and its boiling point is so near that of pure water, that either the molecular weight is enormous—that is, there are but very few molecules present—or the solution is something entirely different from that of sugar, for instance.

The use of the so-called Ultra-microscope by Zsigmondy and Siedentopf in 1903 showed that such solutions were comprised of extremely minute particles in a continual state of movement.

Since this date the great importance of this field has been realised, and in physiological chemistry and in some branches of technical chemistry Colloids are a source of effects so momentous and specific that a new branch of Chemistry has arisen—Colloidal Chemistry—with its own technique and its own journals, devoted to the investigations, relations and laws of a fascinating branch of the science.

**Enzymes.**—Before concluding, I wish to say something about those substances through whose agency, we believe, all life processes take place. In 1836 Berzelius suggested that the chemical action of the living cell was aided by some such bodies as those which brought about catalytic action in the chemical reactions of the laboratory. The value of this suggestion remained unrecognised for nearly seventy years.

Biological Chemistry, as founded by Liebig and his school, occupied itself in the study of the various constituents of the fluids and tissues of the body, the products of cell activity, and the isolation of an enormous number of crystalline chemical individuals. This material, so laboriously collected and studied, together with the technique elaborated for the isolation of such substances, led—as I have indicated—to the synthesis of many of the highly complex and important constituents of animal and plant life.

About 1900 Bredig and his students, working in the Heidelburg laboratories, found methods of preparing colloidal solutions of various metals, and observed that the behaviour of these solutions was so similar to that of the ordinary Enzymes that he christened them the " Inorganic Ferments."

Not only is colloidal platinum—for instance—a most powerful catalyst showing an astonishing power of decomposing hydrogen peroxide, when present in solution to the extent of but one part per million of water, but like the Enzymes of the living cell it is found to be extremely sensitive to the action of heat and poisons. One millionth of a milligram of prussic acid per c.c. reduces the intense action of platinum on hydrogen peroxide to half its original value. The action of one part of sulphuretted hydrogen in 300 millions of water retards that action. This restatement of the similarity of Enzymes and the Catalysts of Chemistry marked the beginning of a great advance in Bio-Chemistry, for it attracted to it the attention of chemists trained in the new conceptions of the science.

The hypothesis that catalytic action in the laboratory was identical with such enzyme action, for instance, as that which results in the oxidation and hydrolysis in the living tissue, incited chemists on the one hand to make a thorough investigation of all the phenomena connected with catalysis and the action of colloidal substances, and on the other hand it gave to Biology a scientific working hypothesis regarding enzyme action. That the vital processes of the body in health and disease are largely dependent upon the presence of Enzymes becomes more and more apparent as these processes are studied, and that the action is catalytic in character explains how such oxidations of Carbon and Hydrogen in the process of respiration and such great chemical changes as those which occur in digestion and assimilation can be carried out so rapidly and at such low temperature in the bodies of animals.

Although some chemical reactions in the laboratory give rise to catalisers during their progress, yet it is a marked characteristic of the living cell to manufacture itself the Enzyme or Enzymes it requires, and this is carried out in a manner quite unknown to us. But apart from this, there does not appear to be any specific difference between the chemistry of the inorganic and organic Enzymes. But in all cases the action of Enzymes in the living body is adapted, controlled and co-ordinated by the cell in a very wonderful fashion, and it should not be forgotten that we have no proof that the similarity between enzyme action and that of inorganic ferments is due to similar causes.

I suppose the ultimate solution of each medical problem lies in the combined attack by a group of investigators each trained in a separate method. Specialisation in science, even in the narrowest sense, is essential to real accomplishment.

But if medical problems are to be solved in this manner, it seems to me that the scientific basis which I have endeavoured to sketch to you should be the foundation of medical studies, and I venture to think the time has come when more time should be found, even in the heavy medical curriculum, for the consideration of those fundamental principles upon which Medicine only can rise to the status of a Science.

That the technique of the science can be mastered is not possible, neither is it necessary, I think, to over-emphasise the value of Organic Chemistry, but what is essential is a thorough understanding of the new armoury of the Science.

